# Improving Physical Activity and Outdoor Recreation in Rural Alabama Through Community Coalitions

**DOI:** 10.5888/pcd16.190062

**Published:** 2019-08-29

**Authors:** William M. Carter, Wayde C. Morse, Ruth W. Brock, Barbara Struempler

**Affiliations:** 1 Alabama Cooperative Extension System, Auburn University, Auburn, Alabama; 2 School of Forestry and Wildlife Sciences, Auburn University, Auburn, Alabama

## Abstract

Obesity rates in the United States are trending upward, and disadvantaged populations continue to have disproportionate rates of obesity. In Alabama, the ALProHealth initiative used community-based participatory research to work with community coalitions to implement research-based interventions that addressed issues related to the lack of opportunities for physical activity in 14 counties whose populations are at high risk of obesity. Coalitions developed work plans and timelines for implementing interventions on the basis of issues discussed during focus groups at the beginning of the ALProHealth initiative. These 14 coalitions implemented 101 interventions related to physical activity in 16 communities. In this evaluation, we measured potential reach and improvements in amenities. The largest reach for an intervention was achieved through marketing and communication efforts, while the most popular intervention, undertaken by the largest number of communities, centered on installing or repairing playground equipment at community parks. Community-based participatory research is an effective method for addressing health issues at the local level, as interventions are developed and readily adopted through active partnerships with community leaders and residents.

**Summary**

**What is already known about this topic?**

Obesity is an epidemic in the United States, and certain regions are affected disproportionately in part as a result of built environments. Community-based participatory research ensures that a community’s health needs are assessed appropriately and interventions to address those needs are developed through active partnerships with community leaders and residents.

**What is added by this report?**

Community coalitions in 14 counties in Alabama with rates of adult obesity at 40% or more implemented 101 interventions to address the lack of access to places for safe, affordable physical activity in 16 communities.

**What are the implications for public health practice?**

Physical activity interventions can be evaluated by calculating potential reach on the basis of census data or by directly measuring changes in the numbers and types of physical activity amenities.

## Introduction

The prevalence of obesity in the United States has reached epidemic levels and continues to grow. Thirty years ago, statewide obesity rates in the United States were below 15% (1). In 2013–2014, more than one-third of the adult population in the United States was obese (2). Previous research identified socioeconomic factors, such as race, income, age, and locale as indicators of the overall health of a population (3). For example, among races, African American people have the highest obesity rates (4). Rates of obesity are higher among people with a low income than among people with a high income (5), among older adults than among young adults (4), and among rural residents than among their urban counterparts (5). Compared with the nation, Alabama has a high percentage of African American residents, people and families living in poverty, older adults, and rural residents. Alabama has the fifth highest rate of adult obesity in the nation (36.3%) and the ninth highest rate of obesity among children and teenagers aged 10 to 17 years (18.2%) (6). In 2014, the adult obesity rates were 40% or greater in 14 Alabama counties.

A positive correlation exists between regular physical activity and good health. Physical activity contributes to reductions in obesity, cardiovascular diseases, diabetes, some cancers, anxiety, stress, and depression (7). Many factors influence participation in physical activity, one of which is the availability of physical activity assets in the built environment. The built environment refers to the physical aspects of an environmental site, which can affect physical activity levels of citizens through the existence of activity-friendly routes, such as sidewalks and bicycle paths, or facilities, such as parks and playgrounds. Just as disparities exist in rates of obesity among racial/ethnic minority populations and low-socioeconomic-status groups, disparities also exist in the quality of built environments that support physical activity and access to resources for physical activity (8). Rural environments are at a particular disadvantage because physical activity and active transportation resources such as sidewalks and bicycle paths are severely limited, and these inadequacies contribute to a higher prevalence of poor health outcomes among rural residents than among their urban counterparts (9).

## Purpose and Objectives

To address obesity in Alabama, the Alabama Cooperative Extension System implemented ALProHealth, a community-based obesity reduction and prevention initiative. The program began in October 2014 and was implemented in 14 Alabama counties that have an adult obesity prevalence of 40% or more: Barbour, Bibb, Bullock, Chambers, Coosa, Crenshaw, Cullman, Escambia, Greene, Lowndes, Macon, Pickens, Sumter, and Wilcox. The population in these 14 counties, when compared with national and state populations, has a higher percentage of African American residents, has a lower income, is older, and has a higher percentage of rural residents (Table 1).

**Table 1:** Comparison of Selected Demographic Characteristics in the United States and in 14 High-Obesity^a^ Counties in Alabama^b^

Location	Percentage African American	Median Family Income, $	Median Age, y	Percentage of Population Living in Rural Areas
14 High-obesity counties in Alabama	36.1	44,669	40.0	74.3
Alabama	26.4	56,828	39.0	40.9
United States	12.2	67,871	37.7	19.3

a “High obesity” defined as having a prevalence of obesity ≥40% among adults: Barbour, Bibb, Bullock, Chambers, Coosa, Crenshaw, Cullman, Escambia, Greene, Lowndes, Macon, Pickens, Sumter, and Wilcox counties.

b Data source: US Census Bureau (11).

The overarching goal of the ALProHealth initiative is to prevent and reduce obesity in these 14 high-risk counties. This goal is being pursued through interventions related to 3 strategic areas: 1) education and technical assistance for built environment approaches, 2) a healthy retail food environment, and 3) opportunities for physical activity. Research-based interventions are being implemented to address each strategy. The objective of this study was to evaluate interventions related to increasing access to places for physical activity in these 14 counties.

## Intervention Approach

The ALProHealth initiative used a community-based participatory research approach to maximize the effectiveness of community health assessments and to increase the likelihood that interventions were developed and adopted through active partnerships with community leaders. ALProHealth was conducted as a partnership between local communities and the Alabama Cooperative Extension System. County extension coordinators and regional extension agents in the 14 counties led the community coalitions. Each county developed a coalition consisting of key members of the community, including city officials, school representatives, faith-based leaders, parks and recreation representatives, grocers, and other local residents interested in improving community health.

After coalitions were formed, the study team held focus groups in February and March 2015 with the 14 community coalitions to elicit information from local residents, particularly information on the challenges to maintaining a healthy lifestyle. We chose a focus group format because it encourages dialogue, provides rich text, efficiently elicits a range of ideas, and builds support and buy-in for community-based projects (10).

We organized focus groups to have 10 to 15 participants and last from 1 to 3 hours. A trained facilitator (R.W.B.), using a semistructured questionnaire, led each focus group discussion. The open-ended questions addressed nutrition education (“Where do you receive information about nutrition?”), access to healthy food (“Where do you go to purchase or receive healthy food?”), and opportunities for physical activity (“Where do you go to participate in physical activity?”). We recorded discussions and produced full transcripts for internal use. We placed a large aerial image (36” × 48”) of the community on a wall of the meeting room to help facilitate discussions about locations in the community. As participants identified locations related to the health of their community (eg, parks, schools, other recreation sites, food stores), the facilitator marked these locations on the map. The facilitator was often assisted by a coalition member who was familiar with the community and able to quickly identify locations being discussed.

A trained researcher (W.M.C.) coded each transcript by using NVivo version 10 (QSR International) to develop themes. The primary themes for coding were the 3 intervention strategies. The coder developed other nodes, on the basis of these 3 strategies, to group similarly themed statements. We converted community maps to a digital format by using ArcGIS version 10.4 (Esri). This conversion allowed us to share maps with focus groups and fellow grantees and to disseminate our research.

After the focus groups, we held meetings with each of the 14 coalitions to recommend research-based interventions. These recommendations were tailored according to the issues and information discussed in focus groups. This second meeting provided an opportunity for coalitions to hear ideas for potential interventions before any work plans were developed. The study protocol was approved by the institutional review board of Auburn University.

## Evaluation Methods

For most ALProHealth interventions, our evaluation consisted of estimating the potential reach of an intervention through the use of census data. For example, if a community coalition decided to add outdoor exercise equipment to an existing walking trail or park, we estimated the number of adults in the community who had access to a safe, affordable place for physical activity as a result of the additional equipment. If a playground was added to a park or school for community use, we estimated the number of children who had access to a new location for physical activity.

We calculated potential reach primarily by using estimated population counts of counties in the American Community Survey (11). If an intervention affected the entire county population, then we considered the entire county population to be reached. For age-specific interventions, such as the installation of playground equipment, we considered the population of children aged 14 or younger in the county. If a project was geared toward teenagers and adults, we considered the population for children aged 10 or older. We counted the number and types of physical activity interventions implemented by community coalitions. Determining actual use of amenities was not feasible because of the logistics of having a research team member monitor locations of physical activity (eg, parks, trails) and then extrapolate these data and a lack of resources to execute those tasks.

## Results

The focus groups yielded similar statements about physical activity opportunities from one community to another. Focus group participants discussed primarily the lack of physical activity opportunities and facilities. Participants noted the following: “there’s not that many opportunities here,” “we don’t have the facilities to have a ball club,” “the children don’t have anything to do,” and “the city doesn’t have a place for recreation.” The development of a work plan led to coalitions discussing the possibility of creating new areas or enhancing community spaces for physical activity. Locations of interest included parks, playgrounds, trails, green spaces, and recreation fields.

Other participants noted a lack of awareness of facilities or programs. One stated, “We have a community life center, a walking track, indoor equipment, 2 weight rooms, and we maybe get 5 or 6 [people] most days to utilize the facility.” Coalitions included communication efforts in their work plans to address promoting existing facilities and resources through events such as annual outdoor celebrations or ribbon-cutting ceremonies for new or updated facilities.

Another theme was weather, an especially important topic in the southeastern United States, where high temperatures and humidity persist throughout much of the year. Participants noted the need for “some type of indoor activities center, where you’ve got climate control” and “more access to indoor activities.” This topic led to coalitions discussing the possibility of creating indoor exercise facilities that could be used by the public for free or an affordable fee.

On an individual level, many participants were frank about their lack of motivation or interest in exercise, stating, “motivation is the key” and “the bottom line is exercise has to be fun.” During work plan development, coalitions discussed the implementation of exercise groups and enjoyable programming to increase the attraction of participating in physical activity.

During the first 4 years of the ALProHealth initiative, the 14 community coalitions implemented 101 physical activity interventions in 16 communities (Table 2) to address the topics discussed in focus groups. Many interventions addressed the lack of facilities and resources for physical activity; the most popular projects were the addition of outdoor exercise or fitness equipment and the addition of playground equipment to enhance existing parks and trails. Some coalitions chose to address the challenge of weather and created indoor spaces for physical activity. Other enhancements made to existing parks and trails included adding rest benches, planting shade trees, installing water fountains, and enhancing the safety of spaces with the addition of lighting. Another popular intervention focused on communication efforts to promote existing resources for physical activity. This intervention was implemented through promotional events, such as annual block party celebrations and ribbon-cutting ceremonies, or through the creation of signage and other print resources to identify local places for physical activity.

**Table 2:** Type and Number of Physical Activity Interventions Implemented by Community Coalitions and Potential Reach of Intervention in 14 High-Obesity^a^ Counties in Alabama, 2014–2018

Intervention	No. of Communities Implementing Intervention	Potential Reach for Community Intervention, No. of People
Promote existing resources for physical activity through signage	14	58,667^b^
Enhance safety, aesthetics, and usefulness of community spaces	12	57,111^b^
Install outdoor exercise/fitness equipment	15	53,979^c^
Establish a new or enhance an existing walking/biking trail	9	48,809^b^
Host a promotional kick-off event to highlight resources for physical activity	12	38,555^b^
Establish or support an indoor community fitness center	7	23,524^c^
Establish and support a walking or exercise group	8	14,284^b^
Install or repair playground equipment at community parks	16	8,363^d^
Hire a professional consultant to improve local parks	3	7,711^b^
Create or promote safe routes to walk/bike to school	2	937^e^
Establish or support community or youth sports and activities	3	815^d^

a “High obesity” defined as having a prevalence of obesity ≥40% among adults: Barbour, Bibb, Bullock, Chambers, Coosa, Crenshaw, Cullman, Escambia, Greene, Lowndes, Macon, Pickens, Sumter, and Wilcox counties.

b Estimated total population of communities implementing intervention.

c Estimated population aged ≥10 in communities implementing intervention.

d Estimated population aged ≤14 in communities implementing intervention.

e Total enrollment of schools implementing intervention.

## Implications for Public Health

Results of focus groups comprising members of community coalitions in 14 counties with a prevalence of adult obesity at 40% or more in Alabama indicated environmental challenges to overcoming obesity. ALProHealth used a community-based participatory research model that recognized the coalition members as decision makers and developers of work plans to address these challenges. Community coalitions implemented 101 research-based interventions, based directly on issues discussed in focus groups, to address the lack of opportunities for physical activity. Interventions with the greatest potential reach were those that enhanced the safety, aesthetics, or usefulness of a community space; established a new walking or biking trail or enhanced an existing one; or installed outdoor exercise or fitness equipment.

Our evaluation of these interventions consisted primarily of calculating potential reach on the basis of estimated population counts. Potential reach is not the strongest measure for determining success; that we used it as a primary measure is a limitation of this study. However, we did not have the resources to collect and extrapolate data on actual use. One future method for counting the number of people using a walking trail is the use of an infrared trail counter. These trail counters could be installed at intervention and control locations to log pre-intervention and post-intervention data.

When planning for physical activity or outdoor recreation interventions at a community level, researchers should consider using a community-based participatory model to increase effectiveness and buy-in for potential interventions. Local knowledge is critical to implementing and sustaining policy, system, or environmental changes, which can be achieved through active partnerships with community leaders.
